# Seminormed double sequence spaces of four-dimensional matrix and Musielak–Orlicz function

**DOI:** 10.1186/s13660-018-1877-6

**Published:** 2018-10-19

**Authors:** Renu Anand, Charu Sharma, Kuldip Raj

**Affiliations:** School of Mathematics, Shri Mata Vaishno Devi University, Katra, India

**Keywords:** Double sequences, Orlicz function, Difference sequences, Seminormed spaces, *n*-normed spaces, 40A05, 40A99, 46A30

## Abstract

In this paper we study seminormed double sequence spaces of a four-dimensional matrix and Musielak–Orlicz function over *n*-normed spaces. We explore some interesting inclusion relations, algebraic and topological properties of these spaces.

## Introduction and preliminaries

Generalizations of single sequence spaces are double sequence spaces which were initially given by Bromwich [2]. Later on, these spaces were investigated by Hardy [13], Móricz and Rhoades [24, 25], Tripathy [39, 40], Başarır and Sonalcan [1] and many other researchers. Hardy [13] presented the idea of regular convergence for double sequences. Recently, Hazarika and Esi [14] studied generalized difference paranormed sequence spaces defined over a seminormed sequence space using ideal convergence. A double sequence $x=(x_{kl})$ is a double infinite array of elements $x_{kl}$ for all $k, l\in\mathbb{N}$. A double sequence has Pringsheim’s limit *L* if, given $\epsilon> 0$, there exists $n\in\mathbb{N}$ such that $|x_{kl} -L|< \epsilon$ whenever $k,l> n $. We shall write it as *P*-$\lim_{k,l\rightarrow\infty}x_{kl}=L$, where *k* and *l* tend to infinity independent of each other. Throughout this paper, the limit of a double sequence means a limit in the Pringsheim’s sense.

Let *w*, $l_{\infty}$, *c* and $c_{0}$ denote the spaces of all, bounded, convergent and null sequences, respectively. Kızmaz [16] explored the concept of difference sequence spaces and studied the difference sequence spaces $l_{\infty}(\Delta)$, $c(\Delta)$ and $c_{0}(\Delta)$. This concept was further explored by Et and Çolak [7] who introduced the spaces $l_{\infty }(\Delta^{m})$, $c(\Delta^{m})$ and $c_{0}(\Delta^{m})$. Let *m* be a nonnegative integer. Then for $Z = c, c_{0}$ and $l_{\infty}$, these sequence spaces are defined as

$$ Z \bigl(\Delta^{m} \bigr) = \bigl\{ x = (x_{k})\in w : \bigl(\Delta^{m}x_{k} \bigr)\in Z \bigr\} , $$

where $\Delta^{m} x = (\Delta^{m} x_{k}) = ( \Delta^{m-1}x_{k} - \Delta^{m-1} x_{k+1})$ and $\Delta^{0} x_{k} = x_{k} $ for all $k \in\mathbb{N}$, which is equivalent to the following binomial representation

$$\Delta^{m}x_{k}=\sum_{v=0}^{m}{\left(-1\right)}^{v}\left(\begin{array}{c} m\\ v \end{array}\right)x_{k+v}.$$

Taking $m = 1$, we obtain the spaces studied by Et and Çolak [7]. Similarly, the difference operators can also be defined on double sequences as

$$\begin{aligned} \Delta x_{k,l} =&(x_{k,l}-x_{k,l+1})-(x_{k+1,l}-x_{k+1,l+1}) \\ =& x_{k,l}-x_{k,l+1}-x_{k+1,l}+x_{k+1,l+1} \end{aligned}$$

and

$$\begin{aligned} \Delta^{m} x_{k,l} = \Delta^{m-1}x_{k,l}- \Delta^{m-1}x_{k,l+1}- \Delta^{m-1}x_{k+1,l}+ \Delta^{m-1}x_{k+1,l+1}. \end{aligned}$$

In [15], Kadak and Mohiuddine extended the notion of an almost convergence and its statistical forms with respect to the difference operator involving the $(p, q)$-gamma function. They estimated the rate of almost convergence of approximating linear operators by means of the modulus of continuity and derived some Voronovskaja type results by using the generalized Meyer–König and Zeller operators. Mohiuddine et al. [21] defined and studied statistical *τ*-convergence, statistical *τ*-Cauchy and $S^{*}(\tau)$-convergence of double sequences in a locally solid Riesz space. Quite recently, Mursaleen and Mohiuddine [28, 29] studied the notion of ideal convergence of double sequences in probabilistic normed spaces and also gave the concept of statistically convergent and statistically Cauchy double sequences in intuitionistic fuzzy normed spaces. For more details also see [22, 23, 30, 38].

In [33], Orlicz introduced functions, now called Orlicz functions, and constructed the sequence space $\ell_{M}$. An Orlicz function $M : [0, \infty) \rightarrow[0, \infty) $ is a continuous, nondecreasing and convex function such that $M(0) = 0$, $M(x)>0$ for $x>0$ and $M(x) \longrightarrow\infty$ as $x \longrightarrow\infty $. The idea of an Orlicz function was used by Lindenstrauss and Tzafriri [18] to define the following sequence space:

$$ \ell_{M} = \Biggl\{ x= (x_{k}) \in w : \sum ^{\infty}_{k=1} M \biggl(\frac{ \vert x_{k} \vert }{\rho} \biggr) < \infty \text{ for some } \rho>0 \Biggr\} , $$

which is known as an Orlicz sequence space. The space $\ell_{M}$ is a Banach space with the norm

$$ \Vert x \Vert = \inf \Biggl\{ \rho> 0 : \sum^{\infty}_{k=1} M \biggl(\frac{ \vert x_{k} \vert }{\rho} \biggr) \leq1 \Biggr\} . $$

A sequence $\mathcal{M} = (M_{k})$ of Orlicz functions is said to be a Musielak–Orlicz function (see [19, 32]). A Musielak–Orlicz function $\mathcal{M}=(M_{k})$ is said to satisfy the $\Delta_{2}$-condition if there exist constants $a, K>0$ and a sequence $c=(c_{k})^{\infty}_{k=1}\in l^{1}_{+}$ (the positive cone of $l^{1}$) such that the inequality

$$ M_{k}(2u)\leq K M_{k}(u) + c_{k} $$

holds for all $k\in\mathbb{N}$ and $u\in\mathbb{R}^{+}$, whenever $M_{k}(u)\leq a$. Recently, Esi [3, 4] introduced some new generalized difference sequence spaces using a modulus function. In [5, 6], Esi et al. constructed new spaces of statistically convergent generalized difference sequences via a modulus function. They studied different properties of such sequences and obtained some inclusion relations involving these new difference sequence spaces.

In the middle of 1960s, Gähler [8] developed a satisfactory theory of 2-normed spaces, while that of *n*-normed spaces can be found in [20]. Since then in the early part of the last century, many researchers studied this concept and acquired various results, see [9–11]. For more details about sequence spaces and *n*-normed spaces, see, for instance, [17, 26, 27, 31, 34–36, 41] and references therein.

Let $A=(a_{mnkl})$ be a four-dimensional infinite matrix of scalars. For all $m,n\in\mathbb{N}$, the sum

$$ y_{mn}=\sum_{k,l=1,1}^{\infty,\infty}a_{mnkl}x_{kl} $$

is called the *A*-mean of the double sequence $(x_{kl})$. A double sequence $(x_{kl})$ is said to be *A*-summable to the limit *L* if the *A*-mean exists for all *m*, *n* in the sense of Pringsheim’s convergence:

$$ P\text{-}\lim_{p,q\to\infty}\sum_{k,l=1,1}^{p,q}a_{mnkl}x_{kl}=y _{mn}\quad \text{and}\quad P\text{-}\lim_{m,n\to\infty}y_{mn}=L. $$

### Theorem 1.1

(Robison [37] and Hamilton [12])

*The four*-*dimensional matrix* *A* *is RH*-*regular if and only if*

(RH_1_): $P\text{-} \lim_{m,n}a_{mnkl}=$ *for each* *k* *and* *l*,
(RH_2_): $P\text{-} \lim_{m,n}\sum_{k,l}|a_{mnkl}|=1$,
(RH_3_): $P\text{-} \lim_{m,n}\sum_{k}|a_{mnkl}|=0$ *for each* *l*,
(RH_4_): $P\text{-} \lim_{m,n}\sum_{l}|a_{mnkl}|=0$ *for each* *k*,
(RH_5_): $\sum_{k,l}|a_{mnkl}|<\infty$ *for all* $m,n\in\mathbb{N}$.

Let $P_{rs}$ denote the class of all subsets of $\mathbb{N}\times \mathbb{N}$ not containing more than $(r,s)$ elements and let $\{\phi_{mn}\}$ denote a nondecreasing double sequence of positive real numbers such that $(m,n)\phi_{m+1,n+1}\leq(m+1),(n+1)\phi_{m,n}$ for all $(m,n)\in\mathbb{N}\times\mathbb{N}$. Let $w''(X)$ and $l_{\infty}''(X)$ denote the spaces of all double and all double bounded sequences, respectively, with elements in *X*, where $(X,q)$ denotes a seminormed space. By $\overline{\theta}=(\theta, \theta,\theta,\dots)$ we denote the zero sequence, where *θ* is the zero element of *X*.

Let $\mathcal{M} = (M_{kl})$ be a Musielak–Orlicz function, $p=(p_{kl})$ a bounded double sequence of positive real numbers, and $u=(u_{kl})$ a double sequence of positive real numbers. Let $(X, \|\cdot,\ldots,\cdot\|)$ be an *n*-normed space and let $A=(a_{mnkl})$ be a nonnegative four-dimensional bounded-regular matrix. Now we define the following classes of sequences:

$$\begin{aligned}& l_{\infty}^{\prime\prime } \bigl[\mathcal{M},A,\Delta^{m},u,p,q, \Vert \cdot,\ldots, \cdot \Vert \bigr] \\& \quad = \Biggl\{ x=(x_{kl})\in w''(X): \sup _{k,l\geq1} \sum^{\infty,\infty}_{k,l =1,1} a_{mnkl} M_{kl} \biggl[q_{kl} \biggl( \biggl\Vert \frac{u_{kl} \Delta^{m} x_{kl}}{\varrho},z_{1},\dots,z_{n-1} \biggr\Vert \biggr) \biggr]^{p_{kl}} < \infty, \\& \qquad \text{for some } \varrho>0 \Biggr\} \end{aligned}$$

and

$$\begin{aligned}& m^{\prime\prime } \bigl[\mathcal{M},A,\Delta^{m},u,\phi,p,q, \Vert \cdot,\ldots, \cdot \Vert \bigr] \\& \quad = \biggl\{ x=(x_{kl})\in w''(X): \sup _{r,s\geq1, \sigma\in P_{rs}} \frac{1}{\phi_{rs}} \sum_{k,l \in\sigma} a_{mnkl} M_{kl} \biggl[ q_{kl} \biggl( \biggl\Vert \frac{u _{kl} \Delta^{m} x_{kl}}{\varrho}, z_{1},\dots,z_{n-1} \biggr\Vert \biggr) \biggr]^{p_{kl}} \\& \qquad < \infty, \text{for some } \varrho>0 \biggr\} . \end{aligned}$$

Throughout the paper, we shall use the following inequality: If $0\leq p_{kl}\leq\sup p_{kl}=H$, $K= \max(1,2^{H-1})$ then

1.1 $$ \vert a_{kl}+b_{kl} \vert ^{p_{kl}}\leq K \bigl( \vert a_{kl} \vert ^{p_{kl}}+ \vert b_{kl} \vert ^{p_{kl}} \bigr) $$

for all $k,l \in\mathbb{N}$ and $a_{kl}, b_{kl}\in\mathbb{C}$. Also $|a|^{p_{kl}}\leq\max(1,|a|^{H})$ for all $a\in\mathbb{C}$.

The main aim of this paper is to study some classes of seminormed double sequences of a four-dimensional matrix by using a Musielak–Orlicz function. Some interesting topological properties and interrelations are also examined.

## Main results

### Theorem 2.1

*Let* $\mathcal{M}=(M_{kl})$ *be a Musielak–Orlicz function*, $p=(p_{kl})$ *a double sequence of positive real numbers*, *and* $u=(u_{kl})$ *a double sequence of positive real numbers*. *Then the sequence spaces* $m^{\prime\prime }[\mathcal{M},A,\Delta^{m},u,\phi,p,q,\|\cdot,\ldots ,\cdot \|]$ *and* $l_{\infty}^{\prime\prime }[\mathcal{M},A,\Delta^{m},u,p,q,\| \cdot, \ldots,\cdot\|]$ *are linear spaces over the complex field* $\mathbb{C}$.

### Proof

We shall prove the assertion for $m^{\prime\prime }[\mathcal{M},A,\Delta^{m},u, \phi,p,q,\|\cdot,\ldots,\cdot\|]$ only. Let $x=(x_{kl})$ and $y=(y_{kl}) \in m^{\prime\prime }[\mathcal{M},A,\Delta^{m},u,p,q,\|\cdot ,\ldots ,\cdot\|]$ and $\alpha, \beta\in\mathbb{C}$. Then there exist positive real numbers $\varrho_{1}$, $\varrho_{2}>0$ such that

$$ \sup_{r,s\geq1, \sigma\in P_{rs}} \frac{1}{\phi_{rs}} \sum _{k,l \in\sigma} a_{mnkl} M_{kl} \biggl[ q_{kl} \biggl( \biggl\Vert \frac{u _{kl} \Delta^{m} x_{kl}}{\varrho_{1}},z_{1}, \dots,z_{n-1} \biggr\Vert \biggr) \biggr]^{p_{kl}} < \infty $$

and

$$ \sup_{r,s\geq1, \sigma\in P_{rs}} \frac{1}{\phi_{rs}} \sum _{k,l \in\sigma} a_{mnkl} M_{kl} \biggl[ q_{kl} \biggl( \biggl\Vert \frac{u _{kl} \Delta^{m} y_{kl}}{\varrho_{2}},z_{1}, \dots,z_{n-1} \biggr\Vert \biggr) \biggr]^{p_{kl}} < \infty. $$

Define $\varrho_{3} = \max(2 |\alpha| \varrho_{1}, 2 |\beta| \varrho_{2})$. Since $\|\cdot,\ldots,\cdot\|$ is an *n*-norm on *X* and $(M_{kl})$ is a nondecreasing and convex function, by using inequality (1.1), we have

$$\begin{aligned}& \sup_{r,s\geq1, \sigma\in P_{r,s}} \frac{1}{\phi_{rs}} \sum _{k,l \in\sigma} a_{mnkl} M_{kl} \biggl[ q_{kl} \biggl( \biggl\Vert \frac{u _{kl} \Delta^{m} (\alpha x_{kl} + \beta y_{kl})}{\varrho_{3}},z_{1}, \dots,z_{n-1} \biggr\Vert \biggr) \biggr]^{p_{kl}} \\& \quad \leq \sup_{r,s\geq1, \sigma\in P_{rs}} \frac{1}{\phi_{rs}} \sum _{k,l \in\sigma} a_{mnkl} M_{kl} \biggl[ q_{kl} \biggl( \biggl\Vert \frac{u _{kl} \Delta^{m} \alpha x_{kl}}{\varrho_{3}},z_{1}, \dots,z_{n-1} \biggr\Vert \biggr) \\& \qquad {}+q_{kl} \biggl( \biggl\Vert \frac{u_{kl} \Delta^{m} \beta y_{kl}}{\varrho _{3}},z_{1}, \dots,z_{n-1} \biggr\Vert \biggr) \biggr]^{p_{kl}} \\& \quad \leq K \sup_{r,s\geq1, \sigma\in P_{rs}} \frac{1}{\phi_{rs}} \sum _{k,l \in\sigma} \frac{1}{2^{pkl}}a_{mnkl} M_{kl} \biggl[ q_{kl} \biggl( \biggl\Vert \frac{u_{kl} \Delta^{m} \alpha x_{kl}}{\varrho_{1}},z _{1},\dots,z_{n-1} \biggr\Vert \biggr) \biggr]^{p_{kl}} \\& \qquad {}+ K \sup_{r,s\geq1, \sigma\in P_{rs}} \frac{1}{\phi_{rs}} \sum _{k,l \in\sigma}\frac{1}{2^{pkl}} a_{mnkl} M_{kl} \biggl[ q_{kl} \biggl( \biggl\Vert \frac{u_{kl} \Delta^{m} \beta y_{kl}}{\varrho_{2}},z_{1}, \dots,z_{n-1} \biggr\Vert \biggr) \biggr]^{p_{kl}} \\& \quad \leq K \sup_{r,s\geq1, \sigma\in P_{rs}} \frac{1}{\phi_{rs}} \sum _{k,l \in\sigma} a_{mnkl} M_{kl} \biggl[ q_{kl} \biggl( \biggl\Vert \frac{u _{kl} \Delta^{m}x_{kl}}{\varrho_{1}},z_{1}, \dots,z_{n-1} \biggr\Vert \biggr) \biggr]^{p_{kl}} \\& \qquad {}+ K \sup_{r,s\geq1, \sigma\in P_{rs}} \frac{1}{\phi_{rs}} \sum _{k,l \in\sigma} a_{mnkl} M_{kl} \biggl[ q_{kl} \biggl( \biggl\Vert \frac{u _{kl} \Delta^{m} y_{kl}}{\varrho_{2}},z_{1}, \dots,z_{n-1} \biggr\Vert \biggr) \biggr]^{p_{kl}} \\& \quad < \infty. \end{aligned}$$

Thus, $\alpha x + \beta y \in m^{\prime\prime }[\mathcal{M},A,\Delta^{m},u, \phi,p,q,\|\cdot,\ldots,\cdot\|]$. Hence, $m^{\prime\prime }[\mathcal{M},A, \Delta^{m},u,p,q,\|\cdot,\ldots,\cdot\|]$ is a linear space. □

### Theorem 2.2

*Let* $\mathcal{M}=(M_{kl})$ *be a Musielak–Orlicz function*, $p=(p_{kl})$ *a bounded sequence of positive real numbers*, *and* $u=(u_{kl})$ *a sequence of positive real numbers*. *Then the space* $m^{\prime\prime }[\mathcal {M},A,\Delta ^{m},u,\phi,p,q,\|\cdot,\ldots,\cdot\|]$ *is a seminormed space with the seminorm* *g* *defined by*

$$\begin{aligned}& g(x)= \inf \biggl\{ (\varrho)^{\frac{p_{kl}}{G}}>0: \\& \hphantom{g(x)=}{} \biggl( \sup_{r,s\geq1, \sigma\in P_{r,s}} \frac{1}{\phi_{rs}} \sum _{k,l \in\sigma} a_{mnkl} M_{kl} \biggl[ q_{kl} \biggl( \biggl\Vert \frac{u _{kl} \Delta^{m} x_{kl}}{\varrho},z_{1}, \dots,z_{n-1} \biggr\Vert \biggr) \biggr]^{p_{kl}} \biggr)^{\frac{1}{G}}\leq1 \biggr\} , \end{aligned}$$

*where* $G= \max\{1, \sup p_{kl}<\infty\}$.

### Proof

Clearly, $g(x)\geq0$ for $x=(x_{kl}) \in m^{\prime\prime }[\mathcal {M},A,\Delta ^{m},u,\phi,p,q,\|\cdot,\ldots,\cdot\|]$. Since $M_{kl}(0)=0$, we get $g(\overline{\theta})=0$. Let $\varrho_{1}>0$ and $\varrho_{2}>0$ be such that

$$ \biggl( \sup_{r,s\geq1, \sigma\in P_{rs}} \frac{1}{\phi_{rs}} \sum _{k,l \in\sigma} a_{mnkl} M_{kl} \biggl[ q_{kl} \biggl( \biggl\Vert \frac{u _{kl} \Delta^{m} x_{kl}}{\varrho_{1}},z_{1}, \dots,z_{n-1} \biggr\Vert \biggr) \biggr]^{p_{kl}} \biggr)^{\frac{1}{G}}\leq1 $$

and

$$ \biggl( \sup_{r,s\geq1, \sigma\in P_{rs}} \frac{1}{\phi_{rs}} \sum _{k,l \in\sigma} a_{mnkl} M_{kl} \biggl[ q_{kl} \biggl( \biggl\Vert \frac{u _{kl} \Delta^{m} x_{kl}}{\varrho_{2}},z_{1}, \dots,z_{n-1} \biggr\Vert \biggr) \biggr]^{p_{kl}} \biggr)^{\frac{1}{G}}\leq1. $$

Let $\varrho= \varrho_{1}+\varrho_{2}$. Then we have

$$\begin{aligned}& \biggl( \sup_{r,s\geq1, \sigma\in P_{rs}} \frac{1}{\phi_{rs}} \sum _{k,l \in\sigma} a_{mnkl} M_{kl} \biggl[ q_{kl} \biggl( \biggl\Vert \frac{u _{kl} \Delta^{m} (x_{kl}+y_{kl})}{\varrho},z_{1}, \dots,z_{n-1} \biggr\Vert \biggr) \biggr]^{p_{kl}} \biggr)^{\frac{1}{G}} \\& \quad = \biggl( \sup_{r,s\geq1, \sigma\in P_{rs}} \frac{1}{\phi_{rs}} \sum _{k,l \in\sigma} a_{mnkl} M_{kl} \biggl[ q_{kl} \biggl( \biggl\Vert \frac{u _{kl} \Delta^{m} (x_{kl}+y_{kl})}{\varrho_{1}+\varrho_{2}},z_{1}, \dots,z_{n-1} \biggr\Vert \biggr) \biggr]^{p_{kl}} \biggr)^{\frac{1}{G}} \\& \quad \leq \biggl( \sup_{r,s\geq1, \sigma\in P_{rs}} \frac{1}{\phi_{rs}} \sum _{k,l \in\sigma} \biggl\{ \biggl(\frac{\varrho}{\varrho _{1}+\varrho _{2}} \biggr) a_{mnkl} M_{kl} \biggl[ q_{kl} \biggl( \biggl\Vert \frac{u_{kl} \Delta^{m} x_{kl}}{\varrho_{1}},z_{1},\dots,z_{n-1} \biggr\Vert \biggr) \biggr]^{p_{kl}} \\& \qquad {}+ \biggl(\frac{\varrho}{\varrho_{1}+\varrho_{2}} \biggr) a_{mnkl} M_{kl} \biggl[ q_{kl} \biggl( \biggl\Vert \frac{u_{kl} \Delta^{m} y_{kl}}{\varrho_{2}},z _{1},\dots,z_{n-1} \biggr\Vert \biggr) \biggr]^{p_{kl}} \biggr\} \biggr)^{ \frac{1}{G}} \\& \quad \leq \biggl(\frac{\varrho}{\varrho_{1}+\varrho_{2}} \biggr) \biggl( \sup_{r,s\geq1, \sigma\in P_{rs}} \frac{1}{\phi_{rs}} \sum_{k,l \in\sigma}a_{mnkl} M_{kl} \biggl[ q_{kl} \biggl( \biggl\Vert \frac{u _{kl} \Delta^{m} x_{kl}}{\varrho_{1}},z_{1},\dots,z_{n-1} \biggr\Vert \biggr) \biggr]^{p_{kl}} \biggr)^{\frac{1}{G}} \\& \qquad {}+ \biggl(\frac{\varrho}{\varrho_{1}+\varrho_{2}} \biggr) \biggl( \sup_{r,s\geq1, \sigma\in P_{rs}} \frac{1}{\phi_{rs}} \sum_{k,l \in\sigma}a_{mnkl} M_{kl} \biggl[ q_{kl} \biggl( \biggl\Vert \frac{u _{kl} \Delta^{m} y_{kl}}{\varrho_{2}},z_{1},\dots,z_{n-1} \biggr\Vert \biggr) \biggr]^{p_{kl}} \biggr)^{\frac{1}{G}} \\& \quad \leq 1. \end{aligned}$$

Since $\varrho's$ are nonnegative, we have

$$\begin{aligned}& g(x + y) \\& \quad = \inf \biggl\{ (\varrho)^{\frac{p_{kl}}{G}}>0 : \\& \qquad {} \biggl( \sup_{r,s\geq1, \sigma\in P_{rs}} \frac{1}{\phi_{rs}} \sum _{k,l \in\sigma} M_{kl} \biggl[ q_{kl} \biggl( \biggl\Vert \frac{u_{kl} \Delta^{m} (x_{kl} + y_{kl})}{\varrho},z_{1},\dots,z_{n-1} \biggr\Vert \biggr) \biggr]^{p_{kl}} \biggr)^{\frac{1}{G}} \leq1 \biggr\} \\& \quad \leq \inf \biggl\{ (\varrho_{1})^{\frac{p_{kl}}{G}}>0 : \\& \qquad {} \biggl( \sup_{r,s\geq1, \sigma\in P_{rs}} \frac{1}{\phi_{rs}} \sum _{k,l \in\sigma} M_{kl} \biggl[ q_{kl} \biggl( \biggl\Vert \frac{u_{kl} \Delta^{m} x_{kl}}{\varrho_{1}}, z_{1},\dots,z_{n-1} \biggr\Vert \biggr) \biggr]^{p_{kl}} \biggr)^{\frac{1}{G}} \leq1 \biggr\} \\& \qquad {}+ \inf \biggl\{ (\varrho_{2})^{\frac{p_{kl}}{G}}>0 : \\& \qquad {} \biggl( \sup_{r,s\geq1, \sigma\in P_{rs}} \frac{1}{\phi_{rs}} \sum _{k,l \in\sigma} M_{kl} \biggl[ q_{kl} \biggl( \biggl\Vert \frac{u_{kl} \Delta^{m} y_{kl}}{\varrho_{2}}, z_{1},\dots,z_{n-1} \biggr\Vert \biggr) \biggr]^{p_{kl}} \biggr)^{\frac{1}{G}} \leq1 \biggr\} \\& \quad = g(x) + g(y). \end{aligned}$$

Thus, $g(x + y) \le g(x) + g(y)$.

Finally, we need to prove that the scalar multiplication is continuous. Let *μ* be any complex number. By definition,

$$\begin{aligned}& g(\mu x) \\& \quad = \inf \biggl\{ (\varrho)^{\frac{p_{kl}}{G}}>0: \\& \qquad {} \biggl( \sup_{r,s\geq1, \sigma\in P_{rs}} \frac{1}{\phi_{rs}} \sum _{k,l \in\sigma} M_{kl} \biggl[ q_{kl} \biggl( \biggl\Vert \frac{ \mu u _{kl} \Delta^{m} x_{kl}}{\varrho},z_{1},\dots,z_{n-1} \biggr\Vert \biggr) \biggr]^{p_{kl}} \biggr)^{\frac{1}{G}} \leq1 \biggr\} \\& \quad = \inf \biggl\{ \bigl( \vert \mu \vert a \bigr)^{\frac{p_{kl}}{G}}>0: \\& \qquad {} \biggl( \sup_{r,s\geq1, \sigma\in P_{rs}} \frac{1}{\phi_{rs}} \sum _{k,l \in\sigma} M_{kl} \biggl[ q_{kl} \biggl( \biggl\Vert \frac{u_{kl}\Delta ^{m}x_{kl}}{a},z_{1},\dots,z_{n-1} \biggr\Vert \biggr) \biggr]^{p_{kl}} \biggr)^{ \frac{1}{G}} \leq1 \biggr\} \\& \quad = \vert \mu \vert \inf \biggl\{ (a)^{\frac{p_{kl}}{G}}>0: \\& \qquad {} \biggl( \sup_{r,s\geq1, \sigma\in P_{rs}} \frac{1}{\phi_{rs}} \sum _{k,l \in\sigma} M_{kl} \biggl[ q_{kl} \biggl( \biggl\Vert \frac{ u_{kl} \Delta^{m}x_{kl}}{a},z_{1},\dots,z_{n-1} \biggr\Vert \biggr) \biggr]^{p_{kl}} \biggr)^{\frac{1}{G}} \leq1, \\& \qquad {} \text{where } a= \frac{\varrho}{ \vert \mu \vert } \biggr\} \\& \quad = \vert \mu \vert g(x). \end{aligned}$$

Thus, the scalar multiplication is continuous. The proof is complete. □

### Proposition 2.3

*For any Musielak–Orlicz function* $\mathcal{M}=(M_{kl})$, *let* $p=(p_{kl})$ *be a bounded sequence of positive real numbers and* $u=(u_{kl})$ *a sequence of positive real numbers*. *Then the space* $l^{\prime\prime }_{\infty}[\mathcal{M},A,\Delta^{m},u,p,q,\|\cdot,\ldots, \cdot\|]$ *is a seminormed space*, *with a seminorm given by*

$$ g(x)= \inf \Biggl\{ (\varrho)^{\frac{p_{kl}}{G}}>0: \sup_{r,s\geq1} \sum _{k,l =1,1}^{\infty, \infty} a_{mnkl} M_{kl} \biggl[ q_{kl} \biggl( \biggl\Vert \frac{u_{kl} \Delta^{m} x_{kl}}{\varrho},z_{1}, \dots,z_{n-1} \biggr\Vert \biggr) \biggr]^{p_{kl}}\leq1 \Biggr\} . $$

### Theorem 2.4

*Let* $\mathcal{M}=(M_{kl})$ *be a Musielak–Orlicz function*. *Then*

$$ m^{\prime\prime } \bigl[\mathcal{M},A,\Delta^{m},u, \phi^{*},p,q, \Vert \cdot,\ldots, \cdot \Vert \bigr] \subset m^{\prime\prime } \bigl[ \mathcal{M},A,\Delta^{m},u,\phi ^{**},p,q, \Vert \cdot, \ldots,\cdot \Vert \bigr] $$

*if and only if* $\sup_{r,s\geq1} \frac{\phi_{rs}^{*}}{\phi_{rs}^{**}} < \infty$ *for all* $r,s \in\mathbb{N}$.

### Proof

Let $x \in m^{\prime\prime }[\mathcal{M},A,\Delta^{m},u,\phi^{*},p,q,\| \cdot, \ldots,\cdot\|]$ and $S = \sup_{r,s\geq1} \frac{\phi_{rs}^{*}}{\phi_{rs}^{**}} < \infty$. Then, we obtain

$$\begin{aligned}& \sup_{r,s\geq1, \sigma\in P_{rs}} \frac{1}{\phi^{**}_{rs}} \sum _{k,l \in\sigma} a_{mnkl} M_{kl} \biggl[ q_{kl} \biggl( \biggl\Vert \frac{u _{kl} \Delta^{m} x_{kl}}{\varrho},z_{1}, \dots,z_{n-1} \biggr\Vert \biggr) \biggr]^{p_{kl}} \\& \quad \leq \sup_{r,s\geq1} \frac{\phi_{rs}^{*}}{\phi_{rs}^{**}} \sup _{r,s\geq1, \sigma\in P_{rs}} \frac{1}{\phi^{*}_{rs}} \sum_{k,l \in\sigma} a_{mnkl} M_{kl} \biggl[ q_{kl} \biggl( \biggl\Vert \frac{u _{kl} \Delta^{m} x_{kl}}{\varrho},z_{1},\dots,z_{n-1} \biggr\Vert \biggr) \biggr]^{p_{kl}} \\& \quad = S \sup_{r,s\geq1, \sigma\in P_{rs}} \frac{1}{\phi^{*}_{rs}} \sum _{k,l \in\sigma} a_{mnkl} M_{kl} \biggl[ q_{kl} \biggl( \biggl\Vert \frac{u _{kl} \Delta^{m} x_{kl}}{\varrho},z_{1}, \dots,z_{n-1} \biggr\Vert \biggr) \biggr]^{p_{kl}} \\& \quad < \infty. \end{aligned}$$

Thus, $x \in m^{\prime\prime }[\mathcal{M},A,\Delta^{m},u,\phi^{**},p,q,\| \cdot ,\ldots,\cdot\|]$.

Conversely, suppose that

$$ m^{\prime\prime } \bigl[\mathcal{M},A,\Delta^{m},u, \phi^{*},p,q, \Vert \cdot,\ldots, \cdot \Vert \bigr] \subset m^{\prime\prime } \bigl[ \mathcal{M},A,\Delta^{m},u,\phi ^{**},p,q, \Vert \cdot, \ldots,\cdot \Vert \bigr] $$

and $x \in m^{\prime\prime }[\mathcal{M},A,\Delta^{m},u,\phi^{*},p,q,\| \cdot, \ldots,\cdot\|]$. Then there exists a $\varrho> 0$ such that

$$ \sup_{r,s\geq1, \sigma\in P_{rs}} \frac{1}{\phi^{*}_{rs}} \sum _{k,l \in\sigma} a_{mnkl} M_{kl} \biggl[ q_{kl} \biggl( \biggl\Vert \frac{u _{kl} \Delta^{m} x_{kl}}{\varrho},z_{1}, \dots,z_{n-1} \biggr\Vert \biggr) \biggr]^{p_{kl}} < \epsilon $$

for every $\epsilon> 0$. Suppose that $\sup_{r,s\geq1} \frac{\phi_{rs}^{*}}{\phi_{rs}^{**}} = \infty$, then there exists a sequence of numbers $(r_{i}, s_{j})$ such that $\lim_{i,j\rightarrow\infty} \frac{\phi_{r_{i}s_{j}}^{*}}{ \phi_{r_{i}s_{j}}^{**}} = \infty$. Hence, we have

$$\begin{aligned}& \sup_{r,s\geq1, \sigma\in P_{rs}} \frac{1}{\phi^{**}_{rs}} \sum _{k,l \in\sigma} a_{mnkl} M_{kl} \biggl[ q_{kl} \biggl( \biggl\Vert \frac{u _{kl} \Delta^{m} x_{kl}}{\varrho},z_{1}, \dots,z_{n-1} \biggr\Vert \biggr) \biggr]^{p_{kl}} \\& \quad \geq \sup_{i,j\geq1} \frac{\phi_{r_{i}s_{j}}^{*}}{\phi_{r_{i}s_{j}}^{**}} \sup _{r,s\geq1, \sigma\in P_{r_{i}s_{j}}} \frac{1}{\phi^{*}_{r_{i}s _{j}}} \sum_{k,l \in\sigma} a_{mnkl} M_{kl} \biggl[q_{kl} \biggl( \biggl\vert \frac{u _{kl} \Delta^{m} x_{kl}}{\varrho},z_{1},\dots,z_{n-1} \biggr\vert \biggr) \biggr]^{p_{kl}} \\& \quad = \infty. \end{aligned}$$

Therefore, $x \notin m^{\prime\prime }[\mathcal{M},A,\Delta^{m},u,\phi ^{**},p,q,\| \cdot,\ldots,\cdot\|]$, which is a contradiction. This completes the proof. □

### Theorem 2.5

*Let* $\mathcal{M}=(M_{kl})$ *be any Musielak–Orlicz function*. *Then*

$$ m^{\prime\prime } \bigl[\mathcal{M},A,\Delta^{m},u, \phi^{*},p,q, \Vert \cdot,\ldots, \cdot \Vert \bigr] = m^{\prime\prime } \bigl[\mathcal{M},A, \Delta^{m},u, \phi^{**},p,q, \Vert \cdot, \ldots,\cdot \Vert \bigr] $$

*if and only if* $\sup_{r,s\geq1} \frac{\phi_{rs}^{*}}{\phi_{rs}^{**}} < \infty$ *and* $\sup_{r,s\geq1} \frac{\phi_{rs}^{**}}{\phi_{rs}^{*}} <\infty$ *for all* $r,s \in\mathbb{N}$.

### Proof

We omit the details since the proof is easy. □

### Theorem 2.6

*For Musielak–Orlicz functions* $\mathcal{M^{\prime}}=(M^{\prime }_{kl})$ *and* $\mathcal{M^{\prime\prime }}=(M^{\prime\prime }_{kl})$ *which satisfy the* $\Delta_{2}$-*condition*, *the following relations hold*:

(i) $m^{\prime\prime } [\mathcal{M},A,\Delta^{m},u,\phi,p,q,\| \cdot, \ldots,\cdot\|] \subset m^{\prime\prime }[\mathcal{M^{\prime}}\circ \mathcal{M^{\prime\prime }},A,\Delta^{m},u,\phi,p,q,\|\cdot,\ldots,\cdot \|]$
(ii) $m^{\prime\prime } [\mathcal{M},A, \Delta^{m},u,\phi,p,q, \Vert \cdot,\ldots ,\cdot \Vert ] \cap m^{\prime\prime } [ \mathcal{M^{\prime\prime }},A, \Delta^{m},u,\phi,p,q, \Vert \cdot, \ldots,\cdot \Vert ] \subset m^{\prime\prime } [\mathcal{M^{\prime}}+ \mathcal{M^{\prime\prime }},A, \Delta^{m},u,\phi,p,q, \Vert \cdot, \ldots,\cdot \Vert ]$.

### Proof

(i) Let $x=(x_{kl}) \in m^{\prime\prime }[\mathcal{M},A,\Delta^{m},u,\phi ,p,q,\| \cdot,\ldots,\cdot\|]$. Then there exists a positive real number $\varrho> 0$ such that

$$ \sup_{r,s\geq1, \sigma\in P_{rs}} \frac{1}{\phi_{rs}} \sum _{k,l \in\sigma} a_{mnkl} M_{kl} \biggl[ q_{kl} \biggl( \biggl\Vert \frac{u _{kl} \Delta^{m} x_{kl}}{\varrho},z_{1}, \dots,z_{n-1} \biggr\Vert \biggr) \biggr]^{p_{kl}} < \infty. $$

Since $\mathcal{M^{\prime}}=(M^{\prime}_{kl})$ is a continuous function, we can find a real number *δ*, $0\le t <\delta$, such that $M^{\prime}_{kl}(t)< \epsilon$. Let $y_{kl}= M^{\prime}_{kl} [q_{kl} ( \| \frac{u _{kl} \Delta^{m} x_{kl}}{\varrho},z_{1},\dots,z_{n-1} \| ) ]$. Hence we can write

$$ \sum_{k,l \in\sigma} a_{mnkl} M^{\prime\prime }_{kl} [ y_{kl} ]^{p_{kl}}= \sum^{\infty}_{y_{kl}\leq\delta} a_{mnkl} M^{\prime\prime }_{kl} [ y_{kl} ]^{p_{kl}} + \sum^{\infty}_{y_{kl}>\delta} a_{mnkl} M^{\prime\prime }_{kl} [ y_{kl} ]^{p_{kl}}, $$

and thus

2.1 $$\begin{aligned} \sum^{\infty}_{y_{kl}\leq\delta} a_{mnkl} M^{\prime\prime }_{kl} [ y_{kl} ]^{p_{kl}} \leq& \max \bigl\{ 1,M^{\prime\prime }_{kl}(1)^{H} \bigr\} \sum ^{\infty}_{y_{kl}\leq\delta} a_{mnkl} [ y_{kl} ]^{p_{kl}}. \end{aligned}$$

For $y_{kl}>\delta$, we use the fact that $y_{kl}<\frac{y_{kl}}{ \delta}<1+\frac{y_{kl}}{\delta}$. By using the definition of $\mathcal{M^{\prime\prime }}=(M^{\prime\prime }_{kl})$, we have

$$ M^{\prime\prime }_{kl} ( y_{kl} )< M^{\prime\prime }_{kl} \biggl(1+ \frac{y_{kl}}{ \delta} \biggr)< \frac{1}{2}M^{\prime\prime }_{kl}(2)+ \frac{1}{2} \biggl(\frac {2y_{kl}}{ \delta} \biggr). $$

Since $\mathcal{M^{\prime\prime }}=(M^{\prime\prime }_{kl})$ satisfies the $\Delta_{2}$-condition and $\frac{y_{kl}}{\delta}>1$, there exists a $T>0$ such that

$$ M^{\prime\prime }_{kl}(y_{kl})< \frac{1}{2} T \frac{y_{kl}}{\delta} M^{\prime\prime }_{kl}(2)+ \frac{1}{2} T \frac{y_{kl}}{\delta} M^{\prime\prime }_{kl}(2)= T \frac{y_{kl}}{ \delta} M^{\prime\prime }_{kl}(2). $$

Therefore, we have

2.2 $$\begin{aligned} \sum^{\infty}_{y_{kl}>\delta} a_{mnkl} \bigl[M^{\prime\prime }_{kl} (y_{kl}) \bigr]^{p _{kl}} \leq& \max \biggl\{ 1, \biggl( \frac{T M^{\prime\prime }_{kl}(2)}{\delta} \biggr)^{H} \biggr\} \sum^{\infty}_{y_{kl}>\delta} a_{mnkl} [y_{kl} ]^{p_{kl}}. \end{aligned}$$

Hence, by inequalities (2.1) and (2.2), we have

$$\begin{aligned}& \sup_{r,s\geq1, \sigma\in P_{rs}} \frac{1}{\phi_{rs}} \sum _{k,l \in\sigma} a_{mnkl} \bigl(M^{\prime}_{kl} \circ M^{\prime\prime }_{kl}\bigr) \biggl[ q _{kl} \biggl( \biggl\Vert \frac{u_{kl} \Delta^{m} x_{kl}}{\varrho},z_{1}, \dots,z_{n-1} \biggr\Vert \biggr) \biggr]^{p_{kl}} \\& \quad = \sup_{r,s\geq1, \sigma\in P_{rs}} \frac{1}{\phi_{rs}} \sum _{k,l \in\sigma} a_{mnkl} \bigl[ M^{\prime\prime }_{kl}( y_{kl}) \bigr]^{p_{kl}} \\& \quad \leq \sup_{r,s\geq1, \sigma\in P_{rs}} \frac{1}{\phi_{rs}}K \sum _{y_{kl}\leq\delta} a_{mnkl}( y_{kl})^{p_{kl}} \\& \qquad {}+ \sup_{r,s\geq1, \sigma\in P_{rs}} \frac{1}{\phi_{rs}} G \sum _{y_{kl}>\delta} a_{mnkl} ( y_{kl})^{p_{kl}}, \end{aligned}$$

where $K= \max\{1,M^{\prime\prime }_{kl}(1)^{H}\}$ and $G= \max\{1, (\frac{TM ^{\prime\prime }_{kl}(2)}{\delta})^{H}\}$.

Hence, $m^{\prime\prime } [\mathcal{M^{\prime}},A,\Delta^{m},u,\phi ,p,q,\|\cdot, \ldots,\cdot\|] \subset m^{\prime\prime }[\mathcal{M^{\prime}}\circ \mathcal{M^{\prime\prime }},A,\Delta^{m},u,\phi,p,q,\|\cdot,\ldots,\cdot \|]$.

(ii) Let

$$ x=(x_{kl}) \in m^{\prime\prime } \bigl[\mathcal{M},A, \Delta^{m},u,\phi,p,q, \Vert \cdot, \ldots,\cdot \Vert \bigr]\cap m^{\prime\prime } \bigl[\mathcal{M^{\prime\prime }},A,\Delta^{m},u, \phi,p,q, \Vert \cdot,\ldots,\cdot \Vert \bigr]. $$

Then

$$ \sup_{r,s\geq1, \sigma\in P_{rs}} \frac{1}{\phi_{rs}} \sum _{k,l \in\sigma} a_{mnkl} M^{\prime}_{kl} \biggl[ q_{kl} \biggl( \biggl\Vert \frac{u _{kl} \Delta^{m} x_{kl}}{\varrho},z_{1}, \dots,z_{n-1} \biggr\Vert \biggr) \biggr]^{p_{kl}} < \infty \quad \text{for some } \varrho> 0 $$

and

$$ \sup_{r,s\geq1, \sigma\in P_{rs}} \frac{1}{\phi_{rs}} \sum _{k,l \in\sigma} a_{mnkl} M^{\prime\prime }_{kl} \biggl[ q_{kl} \biggl( \biggl\vert \frac{u _{kl} \Delta^{m} x_{kl}}{\varrho},z_{1}, \dots,z_{n-1} \biggr\vert \biggr) \biggr]^{p_{kl}} < \infty \quad \text{for some }\varrho> 0. $$

The result follows from the following inequality:

$$\begin{aligned}& \sup_{r,s\geq1, \sigma\in P_{rs}} \frac{1}{\phi_{rs}} \sum _{k,l \in\sigma} a_{mnkl} \bigl(M^{\prime}_{kl} + M^{\prime\prime }_{kl} \bigr) \biggl[ q _{kl} \biggl( \biggl\Vert \frac{u_{kl} \Delta^{m} x_{kl}}{\varrho},z_{1},\dots,z _{n-1} \biggr\Vert \biggr) \biggr]^{p_{kl}} \\& \quad = \sup_{r,s\geq1, \sigma\in P_{rs}} \frac{1}{\phi_{rs}} \sum _{k,l \in\sigma} a_{mnkl} M^{\prime}_{kl} \biggl[ q_{kl} \biggl( \biggl\Vert \frac{u _{kl} \Delta^{m} x_{kl}}{\varrho},z_{1}, \dots,z_{n-1} \biggr\Vert \biggr) \biggr]^{p_{kl}} \\& \qquad {}+ \sup_{r,s\geq1, \sigma\in P_{rs}} \frac{1}{\phi_{rs}} \sum _{k,l \in\sigma} a_{mnkl} M^{\prime\prime }_{kl} \biggl[ q_{kl} \biggl( \biggl\Vert \frac{u _{kl} \Delta^{m} x_{kl}}{\varrho},z_{1}, \dots,z_{n-1} \biggr\Vert \biggr) \biggr]^{p_{kl}} \\& \quad \leq K \sup_{r,s\geq1, \sigma\in P_{rs}} \frac{1}{\phi_{rs}} \sum _{k,l \in\sigma} a_{mnkl} M^{\prime}_{kl} \biggl[ q_{kl} \biggl( \biggl\Vert \frac{u _{kl} \Delta^{m} x_{kl}}{\varrho},z_{1}, \dots,z_{n-1} \biggr\Vert \biggr) \biggr]^{p_{kl}} \\& \qquad {}+ K \sup_{r,s\geq1, \sigma\in P_{rs}} \frac{1}{\phi_{rs}} \sum _{k,l \in\sigma} a_{mnkl} M^{\prime\prime }_{kl} \biggl[ q_{kl} \biggl( \biggl\Vert \frac{u _{kl} \Delta^{m} x_{kl}}{\varrho},z_{1}, \dots,z_{n-1} \biggr\Vert \biggr) \biggr]^{p_{kl}} \\& \quad < \infty, \end{aligned}$$

where $K=\max\{1, 2^{H-1}\}$. Therefore, $x=(x_{kl})\in m^{\prime\prime }[\mathcal{M ^{\prime}}+\mathcal{M^{\prime\prime }},A,\Delta^{m},u,\phi,p,q,\|\cdot ,\ldots, \cdot\|]$. □

### Theorem 2.7

*One has the following inclusions*:

$$ \begin{aligned} l_{1}^{\prime\prime } \bigl[\mathcal{M},A, \Delta^{m},u,p,q, \Vert \cdot,\ldots ,\cdot \Vert \bigr] &\subset m^{\prime\prime } \bigl[\mathcal{M},A,\Delta^{m},u,\phi,p,q, \Vert \cdot ,\ldots ,\cdot \Vert \bigr] \\ &\subset l_{\infty}^{\prime\prime } \bigl[\mathcal{M},A, \Delta^{m},u,p,q, \Vert \cdot, \ldots,\cdot \Vert \bigr], \end{aligned} $$

*where*

$$\begin{aligned}& l_{1}^{\prime\prime } \bigl[\mathcal{M},A,\Delta^{m},u,p,q, \Vert \cdot ,\ldots, \cdot \Vert \bigr] \\& \quad =\Biggl\{ (x_{kl})\in w''(x): \sup _{k,l\geq1} \sum^{\infty,\infty}_{k,l =1,1} a_{mnkl} M_{kl} \biggl[ q_{kl} \biggl( \biggl\Vert \frac{u_{kl} \Delta^{m} x_{kl}}{\varrho},z_{1},\dots,z_{n-1} \biggr\Vert \biggr) \biggr]^{p_{kl}} < \infty, \\& \qquad \textit{for some } \varrho> 0 \Biggr\} . \end{aligned}$$

### Proof

Let $x=(x_{kl}) \in l_{1}^{\prime\prime }[\mathcal{M},A,\Delta^{m},u,p,q,\| \cdot,\ldots,\cdot\|]$. Then

$$ \sup_{k,l\geq1} \sum^{\infty,\infty}_{k,l =1,1} a_{mnkl} M_{kl} \biggl[ q_{kl} \biggl( \biggl\Vert \frac{u_{kl} \Delta^{m} x_{kl}}{\varrho},z_{1},\ldots,z_{n-1} \biggr\Vert \biggr) \biggr]^{p_{kl}} < \infty \quad \text{for some }\varrho> 0. $$

Since $(\phi_{rs})$ is monotonically increasing, it follows that

$$\begin{aligned}& \frac{1}{\phi_{rs}} \sum_{k,l \in\sigma} a_{mnkl} M_{kl} \biggl[ q_{kl} \biggl( \biggl\Vert \frac{u _{kl} \Delta^{m} x_{kl}}{\varrho},z_{1}, \dots,z_{n-1} \biggr\Vert \biggr) \biggr]^{p_{kl}} \\& \quad \leq \frac{1}{\phi_{11}} \sum_{k,l \in\sigma} a_{mnkl} M_{kl} \biggl[ q_{kl} \biggl( \biggl\Vert \frac{u _{kl} \Delta^{m} x_{kl}}{\varrho},z_{1},\dots,z_{n-1} \biggr\Vert \biggr) \biggr]^{p_{kl}} \\& \quad \leq \frac{1}{\phi_{11}} \sum^{\infty,\infty}_{k,l=1,1} a_{mnkl} M_{kl} \biggl[ q_{kl} \biggl( \biggl\Vert \frac{u_{kl} \Delta^{m} x_{kl}}{\varrho},z_{1},\dots,z_{n-1} \biggr\Vert \biggr) \biggr]^{p_{kl}} \\& \quad < \infty. \end{aligned}$$

Thus, $x=(x_{kl}) \in m^{\prime\prime }[\mathcal{M},A,\Delta^{m},u,\phi ,p,q,\| \cdot,\ldots,\cdot\|]$, which implies

$$ l_{1}^{\prime\prime } \bigl[\mathcal{M},A,\Delta^{m},u,p,q, \Vert \cdot,\ldots ,\cdot \Vert \bigr] \subset m^{\prime\prime } \bigl[ \mathcal{M},A,\Delta^{m},u,\phi,p,q, \Vert \cdot ,\ldots, \cdot \Vert \bigr]. $$

Further, let $x=(x_{kl}) \in m^{\prime\prime }[\mathcal{M},A,\Delta ^{m},u,\phi,p,q,\| \cdot,\ldots,\cdot\|]$. Then

$$\begin{aligned}& \sup_{r,s\geq1, \sigma\in P_{rs}} \frac{1}{\phi_{rs}} \sum _{k,l \in\sigma} a_{mnkl} M_{kl} \biggl[ q_{kl} \biggl( \biggl\Vert \frac{u _{kl} \Delta^{m} x_{kl}}{\varrho},z_{1}, \ldots,z_{n-1} \biggr\Vert \biggr) \biggr]^{p_{kl}} < \infty \quad \text{for some }\varrho> 0 \\& \quad \Longrightarrow\quad \sup_{k,l \in\mathbb{N}\times\mathbb{N}} \frac{1}{\phi_{rs}} \sum _{k,l \in\sigma} a_{mnkl} M_{kl} \biggl[ q_{kl} \biggl( \biggl\Vert \frac{u _{kl} \Delta^{m} x_{kl}}{\varrho},z_{1}, \dots,z_{n-1} \biggr\Vert \biggr) \biggr]^{p_{kl}} < \infty \\& \quad \text{for some }\varrho> 0, \end{aligned}$$

where the cardinality of *σ* is taken to be 1. And then also

$$ x=(x_{kl}) \in l_{\infty}^{\prime\prime } \bigl[\mathcal{M},A, \Delta^{m},u,p,q, \Vert \cdot,\ldots,\cdot \Vert \bigr]. $$

Therefore,

$$ m^{\prime\prime } \bigl[\mathcal{M},A,\Delta^{m},u,\phi,p,q, \Vert \cdot,\ldots ,\cdot \Vert \bigr] \subset l_{\infty}^{\prime\prime } \bigl[ \mathcal{M},A,\Delta^{m},u,p,q, \Vert \cdot, \ldots,\cdot \Vert \bigr]. $$

 □
